# Distribution and diversity of diatom assemblages in surficial sediments of shallow lakes in Wapusk National Park (Manitoba, Canada) region of the Hudson Bay Lowlands

**DOI:** 10.1002/ece3.2179

**Published:** 2016-06-09

**Authors:** Olivier Jacques, Frédéric Bouchard, Lauren A. MacDonald, Roland I. Hall, Brent B. Wolfe, Reinhard Pienitz

**Affiliations:** ^1^ Département de géographieUniversité LavalQuébec CityQCCanadaG1V 0A6; ^2^ Centre d'études nordiquesUniversité LavalQuébec CityQCCanadaG1V 0A6; ^3^ Department of BiologyUniversity of WaterlooWaterlooONCanadaN2L 3G1; ^4^ Department of Geography and Environmental StudiesWilfrid Laurier UniversityWaterlooONCanadaN2L 3C5

**Keywords:** Diatoms, Hudson Bay Lowlands, Limnology, Monitoring, Shallow lakes, Wapusk National Park

## Abstract

The hydrology of shallow lakes (and ponds) located in the western Hudson Bay Lowlands (HBL) is sensitive to climate warming and associated permafrost thaw. However, their biological characteristics are poorly known, which hampers effective aquatic ecosystem monitoring. Located in northern Manitoba along the southwestern coast of Hudson Bay, Wapusk National Park (WNP) encompasses numerous shallow lakes representative of the subarctic zone. We analyzed the distribution and diversity of diatom (microscopic algae; class Bacillariophyceae) assemblages in surficial sediments of 33 lakes located in three different ecozones spanning a vegetation gradient, from NE to SW: the Coastal Fen (CF), the Interior Peat Plateau (IPP), and the Boreal Spruce Forest (BSF). We found significant differences (*P* < 0.05) in diatom community composition between CF and IPP lakes, and CF and BSF lakes, but not between IPP and BSF lakes. These results are consistent with water chemistry measurements, which indicated distinct limnological conditions for CF lakes. Diatom communities in CF lakes were generally dominated by alkaliphilous taxa typical of waters with medium to high conductivity, such as *Nitzschia denticula*. In contrast, several IPP and BSF lakes were dominated by acidophilous and circumneutral diatom taxa with preference for low conductivity (e.g., *Tabellaria flocculosa*,* Eunotia mucophila*,* E. necompacta* var. *vixcompacta*). This exploratory survey provides a first detailed inventory of the diatom assemblages in the WNP region needed for monitoring programs to detect changes in shallow lake ecosystems and ecozonal shifts in response to climate variations.

## Introduction

The western Hudson Bay Lowlands (HBL) region in central Canada comprises thousands of shallow lakes and ponds distributed within a permafrost‐influenced landscape. Since the last deglaciation, these freshwater ecosystems have been naturally evolving following the emergence of land in response to ongoing isostatic rebound. However, recent warming in areas such as Wapusk National Park (WNP), located in northeastern Manitoba, has caused the disappearance of permafrost from some southern areas of the park and a 37% increase in the average active‐layer thickness during the past century (Zhang et al. 2012). Accelerated permafrost thaw and climatic changes have resulted in an increasingly dynamic landscape that has variably influenced lake water balance (Wolfe et al. 2011; Bouchard et al. 2013). Permafrost degradation is projected to continue in WNP (Dyke and Sladen 2010; Zhang 2013).

Shallow aquatic systems play key ecological roles in WNP for wildlife, for example, by providing shoreline for polar bear dens and habitat for many migratory birds (Parks Canada 2011). Moreover, shallow lakes and ponds from northern permafrost regions are generally known to be strong emitters of greenhouse gases, namely CO_2_ and CH_4_ (e.g., Laurion et al. 2010; Abnizova et al. 2012). In this context, and with anticipated rapid evolution of these freshwater ecosystems, it is important to explore their limnological and ecological diversity to establish a set of reference conditions. Such knowledge can provide a better understanding of their current status and help anticipate how they will evolve in response to climate changes. Furthermore, such knowledge could be used by WNP managers as a basis for monitoring studies capable of detecting changes in the aquatic landscapes of the park (Parks Canada 2013).

Previous research by Bos and Pellatt (2012) examined the water chemistry of several ponds within and adjacent to WNP and reported gradients in ion concentration related to the proximity of Hudson Bay, and dissolved organic carbon (DOC) concentration related to variation in vegetation density. Other studies have documented the diversity in zooplankton communities and underscored the importance of nutrients in limiting phytoplankton growth (Symons et al. 2012, 2014). Here, we add to the existing knowledge of the freshwater biotic communities of WNP by characterizing the distribution and diversity of their modern diatom (Bacillariophyceae) assemblages. Diatoms are unicellular microscopic photoautotrophs that represent a major component of algal communities. As they are ubiquitous in aquatic environments, taxonomically diverse, occupy specific microhabitats, reproduce rapidly, and are widely distributed, diatoms reflect ecological conditions within an ecosystem and are thus good indicators for tracking variations in water quality and habitat availability (Smol and Stoermer 2010). Their siliceous cell walls (valves) are well preserved in sediment archives, and thus, they can be used to reconstruct limnological conditions in the past (Battarbee et al. 2001). Yet, diatom valves from surficial sediments can also be of great use for monitoring recent environmental changes (the past few years) within lake basins and thus can be cost‐effective for inclusion in National Parks monitoring programs (e.g., Edlund et al. 2013). In addition, diatoms integrate and record prevailing limnological conditions during the growth season, providing more than a single snapshot of limnological conditions at a given moment when samples were collected. This is particularly relevant in the case of shallow lakes and ponds in western HBL, as many experience substantial seasonal water chemistry variations (White et al. 2014). The analysis of the diatom assemblages also allows detection of biological changes that are normally not observed during regular water quality sampling programs (Edlund et al. 2013).

In this study, we analyzed the diatom assemblages in surficial sediments of 33 shallow lakes and ponds (hereafter referred to collectively as lakes) from the WNP region. The lakes cover a broad range of environmental conditions as they are distributed within three different eco‐climatic zones (or ecozones) defined by Parks Canada (2015), from NE to SW: the Coastal Fen (CF), the Interior Peat Plateau (IPP), and the Boreal Spruce Forest (BSF). We aimed to determine whether there were significant differences in diatom community composition across the ecozones and, if so, identify indicator taxa that characterize the differences among them. We assess the potential of diatom assemblages as bioindicators in long‐term monitoring programs to track responses of WNP shallow lakes to warming and associated environmental changes.

## Methods

### Study area

**Figure 1:**
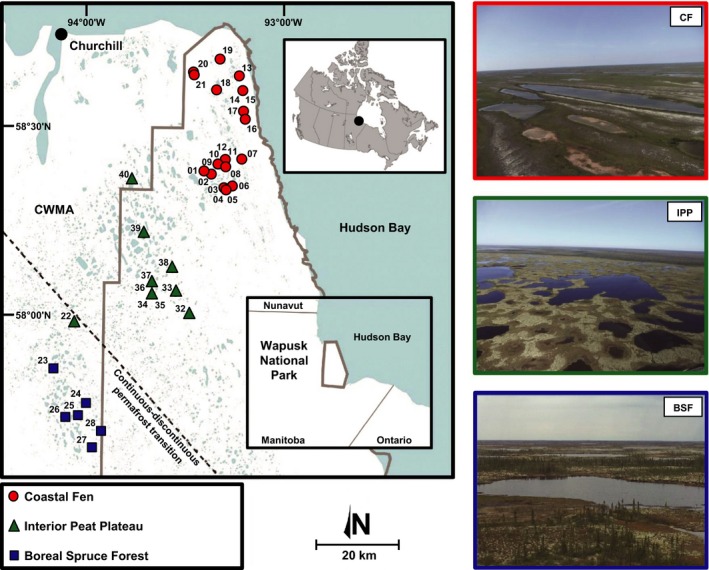
Study area showing the locations of the 37 study lakes located in the Wapusk National Park (Manitoba, Canada) region. Some lakes extend into the Churchill Wildlife Management Area (CWMA) to the southwest. The three ecozones are illustrated at the right of the map (CF: Coastal Fen, IPP: Interior Peat Plateau, BSF: Boreal Spruce Forest).

WNP is located within the western HBL in a low‐relief landscape spanning the continuous–discontinuous permafrost transition (Fig. 1). The underlying bedrock is composed of Precambrian igneous and metamorphic rocks, as well as Paleozoic sedimentary formations of sandstones and carbonates (limestone and dolomite) (Dredge and Nixon 1992). Surface deposits are represented by marine and littoral sands, silts, and clays deposited during the postglacial Tyrrell Sea episode that began after the retreat of the Laurentide Ice Sheet around 8–8.5 ka BP (Dredge and Nixon 1986, 1992; Dyke and Prest 1987). Near the coast, several raised beaches formed by isostatic rebound are separated by water‐filled depressions. The abundance of fine‐grained soils, combined with the prevalence of permafrost, limits hydrological connectivity with groundwater. Water pools on the surface, creating thousands of shallow lakes, many of which are formed by thermokarst processes.

According to climate normals (1981–2010) compiled from the “Churchill A” station (located approximately 35 km west of WNP), mean annual air temperature is −6.5°C, total precipitation is 452.5 mm (of which 176.5 mm, or 40%, falls as snow), and the freezing period extends from mid‐September to mid‐June (Environment Canada 2015). During the ice‐free period, mean monthly air temperature is 7.0°C in June, 12.7°C in July, 12.3°C in August, and 6.4°C in September, and mean monthly rainfall is 41.0 mm in June, 59.8 mm in July, 69.3 mm in August, and 66.0 mm in September. However, recent substantial lake‐level drawdown (and near‐complete desiccation) resulting from lower‐than‐average winter precipitation has been reported for WNP and underscores the importance of snowmelt runoff to maintain lake water balances (Bouchard et al. 2013).

Dominant vegetation in the three ecozones includes sedges and rushes in the CF; moss, lichen, and scattered shrubs in the IPP; and black spruce (*Picea mariana*) in the BSF (Parks Canada 2000). The CF ecozone is also known to be an important breeding ground for the Lesser Snow Goose (*Chen caerulescens caerulescens*) population, which has been identified as a major factor controlling the seasonal carbon cycle and evolution of CF lakes “WAP 20” and “WAP 21” (MacDonald et al. 2014, 2015).

### Surface sediment sampling

In September 2012, 37 lakes were sampled from within the three ecozones (CF: *n *=* *21; IPP: *n *=* *10; BSF: *n *=* *6; Fig. 1). Surface sediments (upper 1–2 cm) were collected using a coring tube (38‐mm internal diameter), transferred into polyethylene bags (*Whirl‐Pak*), and then shipped to the laboratory and stored in the dark at 4°C until analysis.

### Water chemistry data

Limnological measurements and surface water samples were collected from the 37 lakes in July of 2010, 2011, and 2012 and explored in conjunction with the analysis of diatom assemblages in the surficial lake sediments. This time of the year was selected because it likely represents the period of maximum production of diatoms. At each site, a YSI 600QS multimeter was used to collect in situ measurements of pH and conductivity (Cond). Upon return to the field base, water samples were stored in the dark at 4°C until they were passed through a screen (80‐*μ*m mesh) to remove large particles. Water samples were filtered within 12 h of sample collection (cellulose acetate filters: 45‐*μ*m pore size, 47‐mm diameter) prior to determination of concentrations of dissolved inorganic carbon (DIC) and dissolved organic carbon (DOC) by standard analytical methods at Environment Canada's National Laboratory for Environmental Testing (Burlington, Ontario) (Environment Canada 1994). Subsamples of water were acidified (held at 0.02% H_2_SO_4_) and analyzed for total phosphorus (TP) and total Kjeldahl nitrogen (TKN) concentrations at the Biogeochemistry Lab, University of Waterloo following standard methods (TKN = Bran Luebbe, Method No. G‐189‐097; TP = Bran Luebbe, Method No. G‐188‐097; Seal Analytical, Seattle, USA). Alkalinity (Alk) and concentrations of cations and anions were also measured at the Biogeochemistry Lab, University of Waterloo using standard methods (Alk = Bran Luebbe, Method No. G‐148‐95; Seal Analytical, Seattle, USA) and ion chromatography (DIONEX ICS 3000, IonPac AS18 and CS16 analytical columns). Analyzed elements include ammonium (NH_4_), nitrate (NO_3_), fluorine (F), sodium (Na), magnesium (Mg), chlorine (Cl), potassium (K), calcium (Ca), sulfate (SO_4_), and silica (SiO_2_).

### Loss on ignition

Organic matter (% OM) and carbonate (% CaCO_3_) content of the surface sediment samples were estimated by the loss on ignition (LOI) technique, as described by Heiri et al. (2001). For each sample, about 0.5 g of wet sediment was oven‐dried to constant weight (~24 h at 90°C). The remaining dry sediment was then combusted at 550°C for 4 h to remove organic matter and at 950°C for 2 h to remove the carbonate fraction. The organic and carbonate contents (% dry weight) were obtained by weighing samples before and after each combustion step, and evaluating the weight loss.

### Preparation, identification, and enumeration of diatom valves

In the Aquatic Paleoecology Laboratory at Université Laval, sediment samples were freeze‐dried for about 72 h to remove water. For each sample, ~50 mg of dry sediment was placed in a glass vial and 10% hydrochloric acid (HCl) was added to remove carbonates. This solution was removed and replaced by a mixture of sulfuric (H_2_SO_4_) and nitric (HNO_3_) acids and placed in a hot water bath (60°C) for ~2 h to digest organic matter. Then, samples were rinsed several times with distilled water until acid residues were removed. About 1 ml of the resulting diatom solution was dried onto glass coverslips, which were then mounted onto microscope slides using *Naphrax*, a synthetic resin with a high refractive index (Battarbee et al. 2001).

About 500 diatom valves (or between 300 and 500 for lakes showing low abundance; *n *=* *4; WAP 04, WAP 22, WAP 26, and WAP 39) were identified and enumerated along random transects using a Leica DMRB optical microscope. A few samples (*n *=* *4) were not analyzed because of very low diatom abundance (WAP 01, WAP 06, WAP 13, and WAP 24). Identification was carried out to the finest taxonomic level possible (i.e., species or variety/morphotype) at 1000× magnification. Taxonomic identification mainly followed Antoniades et al. (2008), Fallu et al. (2000), Krammer and Lange‐Bertalot (1986, 1988, 1991a,b), and Spaulding et al. (2010). Some taxa were identified using other taxonomic aids (Foged 1981; Lange‐Bertalot and Metzeltin 1996; Krammer 1997a,b, 2000, 2002, 2003; Lange‐Bertalot 2001; Van de Vijver et al. 2004; Levkov 2009; Lange‐Bertalot et al. 2011).

### Numerical and statistical analyses

July water chemistry values collected during years 2010–2012 were averaged for the purpose of the analyses of this study. Some lakes (*n *=* *4) were not considered because of potential bias due to mid‐summer desiccation events during years 2010 and 2012 (WAP 03, WAP 10, WAP 11, and WAP 12). Also, one BSF lake (WAP 28) was excluded because of logistical constraints that prevented annual sampling. Using R Commander version 2.1‐7 (Fox 2005), boxplots were created to illustrate the range and distribution of the water chemistry data within and between each ecozone.

Shannon diversity index (H′) was calculated for all lakes to evaluate diatom biodiversity in surficial sediments, as follows:

$$H^{\prime }=-\sum \left(p_{i}\;\ln p_{i}\right)$$

where *p_i_* is the number of individuals of a given diatom taxon (*i*) divided by the total number of individuals. The taxonomic richness (S), corresponding to the total number of taxa present, was also characterized for each lake.

Using R Commander version 2.1‐7 (Fox 2005), statistical tests were used to determine whether mean values of the water chemistry, LOI, H′, and S variables differed among lakes in the three ecozones (CF, IPP, BSF). Prior to running the tests, we used Shapiro–Wilk and Bartlett tests (*α *= 0.05) to assess whether the variables had a normal distribution and equal variances in the statistical populations, respectively. The variables NH_4_, TP, DIC, Alk, Ca, and H′ did not differ significantly from normal, and variances were statistically equal among the ecozones. Log‐transformations of the variables TKN, F, Mg, Cl, K, SO_4_, SiO_2_, Cond, and % CaCO_3_ were also normally distributed with equal variances. Consequently, differences among ecozones were assessed for these variables (NH_4_, log TKN, TP, DIC, Alk, log F, log Mg, log Cl, log K, Ca, log SO_4_, log SiO_2_, log Cond, log % CaCO_3_, H′) using one‐way ANOVA tests, and Tukey HSD post hoc tests were used to assess pairwise differences between ecozones. In contrast, the variables NO_3_, DOC, pH, Na, % OM, and S could not meet the normal distribution and equality of variance requirements for performing ANOVA tests. Therefore, we used nonparametric Kruskal–Wallis tests followed by post hoc Nemenyi tests to determine whether distributions differed among the ecozones.

The relative abundance of each taxon was computed for all lakes to evaluate composition of diatom communities in surficial sediments. Taxa present at ≥5% abundance in at least one lake were plotted in a relative abundance diagram along with Shannon diversity index and taxonomic richness using the C2 software version 1.7.6 (Juggins 2014). To explore the distribution of diatom community composition among the lakes, we performed a detrended correspondence analysis (DCA) using the software CANOCO version 5.0 (ter Braak and Šmilauer 2012). DCA was appropriate because the first axis captured a long gradient (5.4 SD units) of compositional turnover (Šmilauer and Lepš 2014). Only diatom taxa presenting a relative abundance ≥1% in at least one lake were considered for the analysis. Water chemistry data were entered as “supplementary data” to assess correlations between distributions of diatoms and environmental gradients. The lakes previously eliminated because of potential bias in water chemistry values and logistical constraints (WAP 03, WAP 10, WAP 11, WAP 12, and WAP 28) were not considered in the DCA. An indirect gradient analysis (DCA) was chosen over a direct gradient analysis with limnological data entered as “explanatory variables” (e.g., RDA, CCA) because the dataset was relatively small and the main emphasis for this study was placed on the distribution patterns of community composition. All diatom percent abundances and environmental variables, except pH, were log‐transformed before ordination by DCA.

A one‐way analysis of similarities (ANOSIM) test was performed to determine whether the composition of diatom assemblages in surficial sediments differed significantly among the three ecozones (CF, IPP, BSF). The ANOSIM test statistic (global R) reflects the observed differences in diatom community composition between lakes in the three ecozones, contrasted with the differences among replicates within each ecozone and ranges from 0 to 1. A value of 0 indicates that the similarity between and within ecozones is the same on average. A value of 1 indicates that replicates within an ecozone are more similar to each other than to all other replicates of other ecozones (Clarke et al. 2014). A *P*‐value was computed by comparing the distribution of within‐ and across‐ecozone rank Bray–Curtis similarities (9999 computations) to the initial rank similarity, as reported by the global *R* value (Clarke et al. 2014; Clarke and Gorley 2015). Similarity percentage analysis (SIMPER; Clarke et al. 2014) was then used to identify the diatom taxa that accounted for the greatest observed differences among the three ecozones. Taxa contributing to >2% of the average similarity within a single ecozone and to >2% of the average dissimilarity for each pairwise comparison between ecozones were considered as potential “indicator” taxa that are most representative of an ecozone. These criteria are similar to those used in other recent studies (e.g., Sokal et al. 2008; Wiklund et al. 2010). The ANOSIM test and SIMPER analyses were performed on square‐root transformed percent abundances of all diatoms that could be identified to the species level or finer, using the program PRIMER version 7 (Clarke et al. 2014; Clarke and Gorley 2015). For all statistical tests, alpha was set at 0.05.

## Results

### Water chemistry data

**Figure 2:**
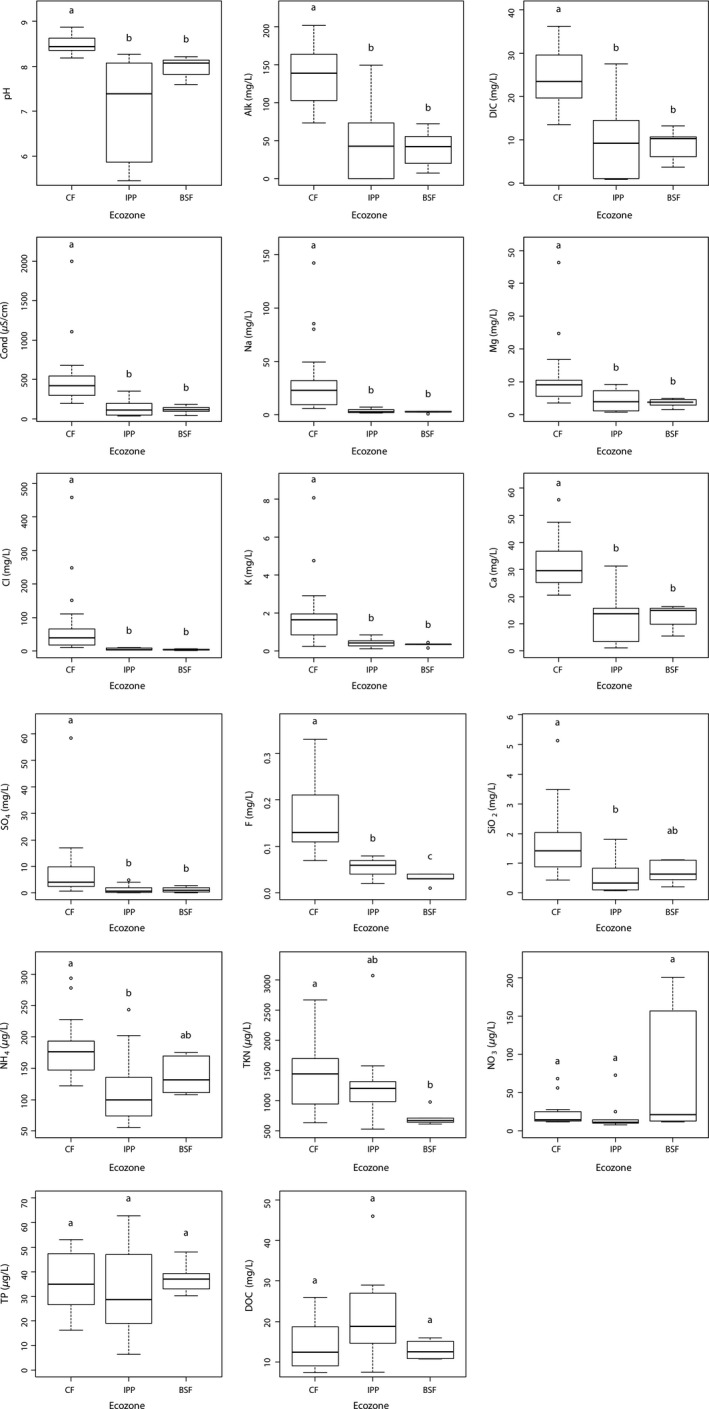
Boxplots illustrating the distribution of the water chemistry variables (July averages for years 2010–2012) in study lakes of the three ecozones from the Wapusk National Park region (CF: Coastal Fen, IPP: Interior Peat Plateau, BSF: Boreal Spruce Forest). The boxes identify the 1st (Q1) and 3rd (Q3) quartiles, and the horizontal bar within each box represents the median value. The whisker bars mark the range of the data, excluding the outliers (empty circles) which are defined as values plotting 1.5 * interquartile range beyond Q1 and Q3. Two ecozones sharing at least one identical letter indicates that their means do not statistically differ.

Water chemistry variables differed among lakes of the three ecozones (Fig. 2; Tables S1 and S2). Values of pH, Alk and Cond, and concentrations of DIC and of most ions (Na, Mg, Cl, K, Ca, SO_4_) were all significantly higher in CF lakes, but did not differ significantly between lakes in the IPP and BSF ecozones. Fluorine concentrations were statistically different between each pair of ecozones, with highest values in CF lakes, intermediate values in IPP lakes, and lowest values in BSF lakes. Significantly higher SiO_2_ and NH_4_ values were found for CF lakes compared to IPP lakes. TKN concentrations differed significantly between lakes in the CF and BSF ecozones. Concentrations of NO_3_, TP, and DOC did not differ significantly among the ecozones.

### Loss on ignition

Means of organic matter and carbonate content did not differ significantly among the three ecozones (Tables S1 and S3). However, important variability in organic matter content could be observed among lakes of the same ecozone, with values ranging from 2% to 90% in CF lakes (median: 76%), from 2% to 93% in IPP lakes (median: 88%), and from 2% to 92% in BSF lakes (median: 81%). Carbonate content varied similarly, ranging from 1% to 72% in CF lakes (median: 12%), from 1% to 29% in IPP lakes (median: 5%), and from 2% to 9% in BSF lakes (median: 4%).

### Distribution of the abundant diatom taxa

**Figure 3:**
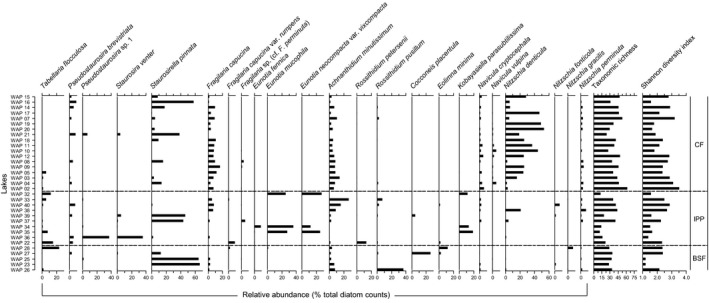
Relative abundance of the diatom taxa frequently found (relative abundance ≥5% in at least one lake) in surficial sediments collected from the study lakes in the Wapusk National Park region. Taxonomic richness and Shannon diversity index are also indicated. Lakes are listed in order of increasing distance from the coast. The dashed lines mark the division between the ecozones (CF: Coastal Fen, IPP: Interior Peat Plateau, BSF: Boreal Spruce Forest).

**Figure 4:**
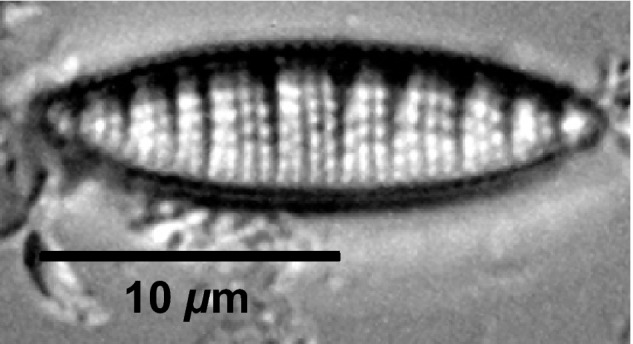
Microscope photograph of diatom species *Nitzschia denticula* (1000×).

In total, 235 diatom taxa belonging to 58 genera were identified in the surface sediment samples from the 33 lakes analyzed (Table S4). The most abundant taxa are shown in Figure 3. Surface sediment assemblages of most CF lakes were dominated by *Nitzschia denticula* (Fig. 4), a species uncommon in the other ecozones. The relative abundance of this taxon was higher than 20% in more than half of CF lakes (10 of 18). The taxa *Fragilaria capucina*,* Navicula cryptocephala*, and *N. vulpina* were also generally more abundant in lakes of the CF ecozone. In contrast, *Tabellaria flocculosa* was abundant in several IPP lakes and one BSF lake, but was rare in most CF lakes. To a lesser extent, *Eolimna minima* and *F. capucina* var. *rumpens* were also better represented in IPP and BSF lakes. The taxa *Eunotia mucophila*,* Eunotia neocompacta* var. *vixcompacta*, and *Kobayasiella parasubtilissima* were almost exclusively found at three IPP lakes (WAP 32, WAP 34, WAP 35) with percentages ranging from 10% to <30%. The taxa *Pseudostaurosira brevistriata*,* Staurosirella pinnata*,* Achnanthidium minutissimum*, and *Nitzschia perminuta* did not show distinctive ecozonal patterns, but rather were commonly found in the lakes from all three ecozones. *Staurosirella pinnata*, in particular, occurred at high relative abundance (>40%) in several lakes. Other taxa were dominant in a single lake, but only found in low relative abundances elsewhere. These include *Staurosira venter* and *Pseudostaurosira* sp. 1 in lake WAP 36 (IPP), *Rossithidium pusillum* in lake WAP 26 (BSF), and *Cocconeis placentula* in lake WAP 27 (BSF).

### Shannon diversity index and richness

Figure 3 presents the Shannon diversity index and taxonomic richness calculated for each lake. Results from the statistical tests indicated a higher taxonomic richness in CF lakes in comparison to IPP lakes, but no significant differences in richness between BSF lakes and lakes from the other ecozones (Table S1). Diversity values did not differ significantly among the ecozones.

### Detrended correspondence analysis

**Figure 5:**
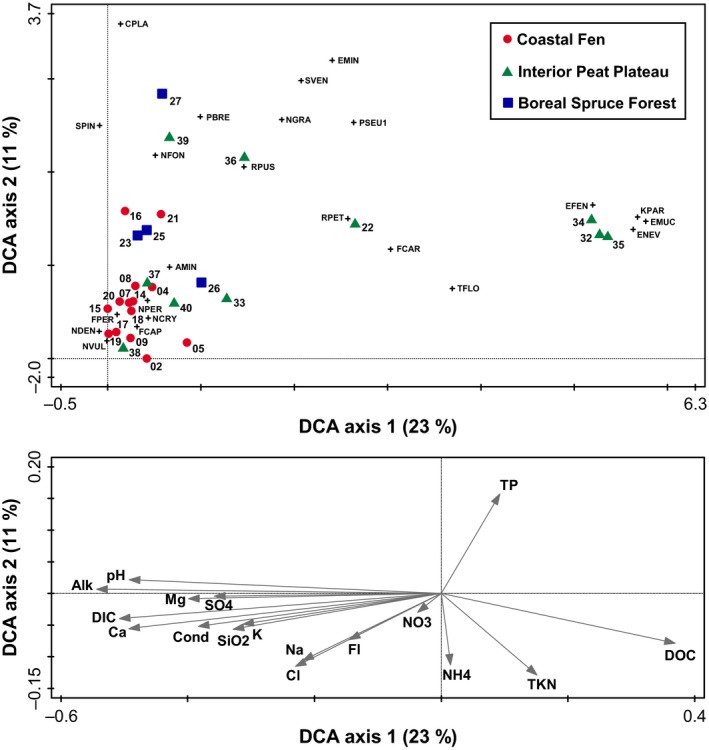
Detrended correspondence analysis (DCA) plots showing the sample (*n *=* *28) and taxon scores (upper panel) and the passively projected environmental gradients (represented by the arrows; lower panel) for the study lakes in the Wapusk National Park region. Only the most abundant diatom taxa (relative abundance ≥5% in at least one lake) are illustrated: AMIN,* Achnanthidium minutissimum*; CPLA,* Cocconeis placentula*; EFEN,* Eunotia fennica*; EMIN,* Eolimna minima*; EMUC,* Eunotia mucophila*; ENEV,* Eunotia neocompacta* var. *vixcompacta*; FCAP,* Fragilaria capucina*; FCAR,* Fragilaria capucina* var. *rumpens*; FPER,* Fragilaria* sp. (cf. *F. perminuta*); KPAR,* Kobayasiella parasubtilissima*; NCRY,* Navicula cryptocephala*; NVUL,* Navicula vulpina*; NDEN,* Nitzschia denticula*; NFON,* Nitzschia fonticola*; NGRA,* Nitzschia gracilis*; NPER,* Nitzschia perminuta*; PBRE,* Pseudostaurosira brevistriata*; PSEU1, *Pseudostaurosira* sp. 1; RPET,* Rossithidium petersenii*; RPUS,* Rossithidium pusillum*; SVEN,* Staurosira venter*; SPIN,* Staurosirella pinnata*; TFLO,* Tabellaria flocculosa*.

DCA axes 1 and 2 explained 23% and 11% of the variability in the diatom taxa data, respectively (Fig. 5). Most of CF lakes plotted close together at the left end of DCA axis 1, associated with relatively high abundance of *N. denticula*,* F. capucina*,* Fragilaria* sp. (cf*. F*. *perminuta*), *N. cryptocephala*,* N. vulpina*, and *N. perminuta*, and relatively high values of lake water pH, Alk, and Cond, and high concentrations of DIC and ions. Sample scores for IPP lakes were broadly distributed across the range of DCA axes 1 and 2, with lakes WAP 37, WAP 38, and WAP 40 plotting within the space occupied by most of the CF lakes. Sample scores for IPP lakes WAP 22, WAP 36, and WAP 39 were located near the middle of axes 1 and 2, whereas sample scores for WAP 32, WAP 34, and WAP 35 were positioned at the right end along axis 1. These latter three lakes were positioned close to species scores for *Eunotia fennica*,* K. parasubtilissima*,* E. mucophila*, and *E. neocompacta* var. *vixcompacta*, suggesting they support high relative abundances of these taxa, and were associated with relatively low lake water pH, Alk, and Cond, high concentration of DOC, and low concentrations of DIC and ions. Sample scores for BSF lakes WAP 23, WAP 25, and WAP 26 positioned close to those for many of CF lakes, and IPP lakes WAP 33, WAP 37, and WAP 40. These lakes were associated with high relative abundance of *A. minutissimum*,* N. perminuta*, and *N. fonticola*. In contrast, BSF lake WAP 27 was positioned alone, high along axis 2 and low on axis 1, and was associated with high relative abundance of *C. placentula*.

### ANOSIM and SIMPER analyses

Results of the ANOSIM test identified that composition of diatom assemblages differed significantly among the three ecozones (*R*‐statistic = 0.529; *P* < 0.0001; 9999 permutations performed). Based on pairwise comparisons, community composition differed significantly between CF and IPP lakes (*R* = 0.552, *P* < 0.0001), and between CF and BSF lakes (*R* = 0.753, *P* < 0.0001), but not between IPP and BSF lakes (*R* = −0.117, *P* = 0.883). SIMPER analysis revealed a few potential indicator diatom taxa (i.e., diatom taxa accounting for >2% of the average similarity in diatom community composition within only one ecozone and to >2% of the average dissimilarity for each pairwise comparison between ecozones). The potential indicator taxa were *N. denticula* for CF lakes; *E. mucophila*,* E. neocompacta* var. *vixcompacta*, and *K. parasubtilissima* for IPP lakes; and *R. pusillum* for BSF lakes (Table 1).

**Table 1:** Results from the SIMPER analysis presenting the diatom taxa contributing to ≥2% of the average similarity in diatom community composition in only one ecozone and to ≥2% of the average dissimilarity for each pairwise comparison between ecozones for the study lakes in the Wapusk National Park region

Diatom taxa	Similarity contribution (%)	Dissimilarity contribution (%) CF‐IPP	Dissimilarity contribution (%) CF‐BSF	Dissimilarity contribution (%) IPP‐BSF
CF				
*Nitzschia denticula*	17.76	7.06	7.64	
IPP				
*Eunotia mucophila*	8.49	3.33		3.85
*Eunotia neocompacta* var. *vixcompacta*	6.04	2.72		3.13
*Kobayasiella parasubtilissima*	5.26	2.27		2.54
BSF				
*Rossithidium pusillum*	6.37		2.90	3.35

CF, Coastal Fen; IPP, Interior Peat Plateau; BSF, Boreal Spruce Forest.

## Discussion

### Limnological characteristics

Among the studied lakes, those belonging to the CF ecozone clearly stood apart with respect to their limnological properties. Several water chemistry variables indeed differed significantly between lakes in the CF and IPP ecozones, and CF and BSF ecozones, but not between IPP and BSF ecozones. CF lakes were associated with alkaline and ion‐rich waters, which contrast with the generally circumneutral (or less alkaline) to acidic pH and low electrolyte content of IPP and BSF lakes. As discussed by Bos and Pellatt (2012), lakes in the CF are located near the Hudson Bay coast and are exposed to more marine‐derived particles (salts) than farther inland, which contributes to higher conductivity/salinity. The effect of airborne sea spray on shallow lakes has frequently been observed in coastal zones, for example, by Pienitz et al. (1997), Dallimore et al. (2000), and Manasypov et al. (2014) in northern Yukon and Northwest Territories (Canada), and western Siberia, respectively. The presence of surficial substrates with carbonate lithology along the coast and in the northernmost part of the park (Dredge and Nixon 1986) probably also accounts for the higher conductivity and pH of CF lakes. In addition, catchment disturbance by lesser snow geese likely enhances erosion and delivery of dissolved constituents in some lakes, such as WAP 20 and WAP 21 (MacDonald et al. 2015). IPP and BSF lakes are generally surrounded by more luxurious vegetation and peat deposits, which probably results in more acidic organic inputs into their waters. The absence of significant differences between IPP and BSF lakes for most of the water chemistry variables likely reflects similarities in the landscape characteristics of these two ecozones. This result may also be attributed to the small sample size within the IPP and BSF ecozones, which may have been insufficient to capture limnological differences.

### Diatom community composition

Surface sediment diatom assemblages in lakes from all ecozones were generally dominated by benthic and epiphytic taxa, although one tychoplanktonic taxon (*T*. *flocculosa*) occurred in high relative abundance in a few lakes. Strictly planktonic taxa (e.g., *Aulacoseira ambigua*,* Aulacoseira distans*,* Discostella stelligera*) were rare, which is consistent with the shallow nature of the lakes. Diatom assemblages were also typical of oligotrophic conditions with low occurrence and relative abundance of diatom taxa indicative of meso‐trophic or eutrophic conditions (e.g., *Asterionella formosa*). Consistent with the results obtained for several of the water chemistry variables, significant differences in diatom community composition were found between CF and IPP lakes, and CF and BSF lakes, but not between IPP and BSF lakes. CF lakes were all dominated by alkaliphilous diatom taxa, which prefer (or are indifferent to) medium to high conductivity, including taxa such as *N. denticula* (Foged 1981; Krammer and Lange‐Bertalot 1988), *S. pinnata* (Foged 1981; Zimmermann et al. 2010), *F. capucina* (Foged 1981; Antoniades et al. 2008), and *N. vulpina* (Lange‐Bertalot 2001). Similarity in diatom community composition among CF lakes reflects the relative uniformity of limnological conditions and catchment characteristics of these lakes. In comparison, IPP and BSF ecozones are characterized by more heterogeneous landscapes and, accordingly, expressed greater variation in diatom community composition. Some lakes from these ecozones resembled CF lakes with dominance by alkaliphilous taxa (e.g., *S. pinnata*). Yet, in contrast to CF lakes, acidophilous and circumneutral diatom taxa with often ecological preferences for low lake water conductivity, such as *T. flocculosa* (Rawson 1956; Germain 1981; Round 1990), *E. mucophila* and *E. necompacta* var. *vixcompacta* (Lange‐Bertalot et al. 2011), and *R. pusillum* (Krammer and Lange‐Bertalot 1991b; Hofmann et al. 2011), were found in high relative abundances in several IPP and BSF lakes. We observed no significant differences in diatom biodiversity among the ecozones and found that taxonomic richness differed only between CF and IPP lakes. We attribute this latter feature to the presence of several low‐pH lakes in the IPP ecozone because acidic conditions are typically associated with lower richness in algal communities (Stokes 1986; Dixit and Smol 1989) as they require specific metabolic and physiological adaptations (Gross 2000).

Among the identified diatom taxa, *N. denticula* demonstrated the clearest differences among ecozones, with high abundance in most CF lakes and lower abundance in IPP and BSF lakes. Differences in pH and conductivity among the ecozones seem to be an important factor associated with the distribution of this species in the shallow lakes of the WNP region. However, other factors, such as characteristics of the catchment areas and benthic substrate type, must also be considered. Indeed, *N. denticula* has been identified to have ecological preferences for plant (Douglas and Smol 1995; Michelutti et al. 2003) and benthic biofilm substrates. Dominance of this species in shallow lakes from Banks Island (NWT, Canada) has been associated with lush catchment areas providing microhabitats rich in mosses and grasses (Lim et al. 2008). It has also been associated with warmer and more nutrient‐rich waters (Lim et al. 2001a,b, 2008). In a more regional context, *N. denticula* has been found in high abundance in benthic biofilms in many ponds located near Churchill, Manitoba (Canada), very near to our study lakes (Macrae 1998; White et al. 2014). The low abundance of *N. denticula* in a few CF lakes despite favorable lake water conditions (i.e., alkaline pH and medium to high conductivity) could, therefore, be attributed to the absence of appropriate substrates for supporting its growth. *Staurosirella pinnata* dominated diatom assemblages in CF lakes with low abundance of *N. denticula* as observed by Macrae (1998) in ponds located near Churchill. *Staurosirella pinnata* is also an alkaliphilous species sometimes found in habitats with moderate to high conductivity (Foged 1981; Zimmermann et al. 2010). However, in contrast to *N. denticula*,* S. pinnata* seems to be generally more dominant on inorganic (sandy or gravely) or muddy sediments than on vegetal substrates (Douglas and Smol 1995; Winter and Duthie 2000; Michelutti et al. 2003; Hofmann et al. 2011). This is supported by paleolimnological analyses from MacDonald et al. (2015), which reported a decrease in relative abundance of *S. pinnata* and an increase in *N. denticula* in CF lakes WAP 20 and WAP 21 that coincided with a change from mineral to more organic sediments.

Another interesting feature in diatom community composition in WNP shallow lakes is displayed by *E. mucophila*,* E. neocompacta* var. *vixcompacta*, and *K. parasubtilissima*, which occurred in high relative abundance in three IPP lakes (WAP 32, WAP 34, and WAP 35) but were almost absent elsewhere. *Eunotia fennica* was also found in notable abundance in IPP lake WAP 34, but was almost absent elsewhere. Lange‐Bertalot et al. (2011) described *E. mucophila* and *E. necompacta* sensu lato as both occurring in abundance in places such as *Sphagnum* peat bogs or acidic dystrophic lakes with low conductivity and nutrient content. *Kobayasiella parasubtilissima* is typically found in similar habitats (e.g., Kulikovskiy et al. 2010; Witkowski et al. 2011; Poulíčková et al. 2013), and *E. fennica* is also commonly associated with *Sphagnum* ponds (Hamilton and Siver 2010; Lange‐Bertalot et al. 2011). All of these taxa have previously been reported from peat surface samples and cores in the southern HBL by Hargan et al. (2015a,b). In particular, high relative abundances of *E. mucophila* and *E. neocompacta* sensu lato have been found in fossil diatom assemblages marking the onset of bog conditions in a core collected from a peatland (Hargan et al. 2015b). In our study, the distinctive diatom communities of IPP lakes WAP 32, WAP 34, and WAP 35 were associated with their particularly low pH. In addition, organic‐rich peat in their catchments provides specific microhabitats, which are also probably favorable to the growth of *Eunotia* taxa. *Eunotia mucophila*, especially, is common in bog hollows in peatlands (e.g., Chen et al. 2012, 2014; Hargan et al. 2015a) and has been associated with particular *Sphagnum* species (e.g., Buczkó 2006). Hargan et al. (2015a), however, suggested that these close associations may be simply attributable to overlap in pH and moisture preferences.

### Implications for biomonitoring

Results from the SIMPER analysis highlighted a few important diatom taxa accounting for differences among the ecozones, namely *N. denticula* for CF lakes; *E. mucophila*,* E. neocompacta* var. *vixcompacta*, and *K. parasubtilisimma* for IPP lakes; and *R. pusillum* for BSF lakes. Yet, the number of discriminating taxa is low and perhaps indicates that shallow lakes from the WNP region support similar microhabitat availability. Given that only *N. denticula* appears to be a uniquely informative indicator of CF lakes, we do not recommend the use of an indicator approach for biomonitoring. Instead, we suggest use of the entire diatom assemblage. For example, the DCA performed in this study integrates information from all diatom taxa that reached at least 1% of relative abundance in at least one lake and could serve as a reference for biomonitoring. In future years, we suggest the sampling of a subset of lakes (minimum 2–3 lakes) in each ecozone, perhaps every 5 years, and analyzing the diatom assemblages in their surficial sediments. The selected lakes should be the most representative of the diversity in diatom community composition among each ecozone (i.e., sample scores broadly distributed across the range of DCA axes 1 and 2). For instance, WAP 02, WAP 16, and WAP 19 could be selected for CF lakes; WAP 22, WAP 32, WAP 37 for IPP lakes; and WAP 23, WAP 26, and WAP 27 for BSF lakes. Passive plotting of the results onto the DCA biplot in Fig. 5 could be employed to detect transitions of lakes in one ecozone toward diatom community composition typical of another ecozone, or staying within the range of its ecozone. Changes in environmental conditions of the lakes of each ecozone could therefore be inferred. For example, a shift of CF sample scores toward the right end along DCA axis 1 would indicate a decrease in lake water pH and/or conductivity and, thus, a transition toward limnological conditions more typical of IPP and BSF ecozones.

## Conclusion

Our study provides a first detailed inventory of modern diatom diversity and distribution in shallow lakes of the Wapusk National Park (Manitoba, Canada) region. We identified significant differences between the diatom community composition of CF and IPP lakes, and CF and BSF lakes, but found no apparent differences between IPP and BSF lakes. The differences in diatom community composition (where observed) are consistent with the prevailing limnological conditions among the ecozones. Integration of surface sediment analysis of diatom community composition for biomonitoring, and relations with water chemistry, could be used to detect consequences of ongoing warming on shallow lake ecosystems of the WNP region.

## Conflict of Interest

None declared.

## Supporting information


**Table S1.** Summary of the statistical tests performed to assess if water chemistry variables differed among lakes of the three ecozones of the Wapusk National Park region.
**Table S2.** Values of the environmental variables obtained for the study lakes in the Wapusk National Park region.
**Table S3.** Loss‐on‐ignition (LOI) results from the surface sediments of the study lakes in the Wapusk National Park region.
**Table S4.** List of the diatoms taxa identified in surficial sediments of the study lakes in the Wapusk National Park region.
