# Supplementary material for: Distribution and diversity of diatom assemblages in surficial sediments of shallow lakes in Wapusk National Park (Manitoba, Canada) region of the Hudson Bay Lowlands
Source: Ecol Evol. 2016 Jun 9;6(13):4526–40. doi: 10.1002/ece3.2179 (PMC4930999; doi:10.1002/ece3.2179)
Supplement: Supplementary file 1 — Table S1. Summary of the statistical tests performed to assess if water chemistry variables differed among lakes of the three ecozones of the Wapusk National Park region. Table S2. Values of the environmental variables obtained for the study lakes in the Wapusk National Park region. Table S3. Loss‐on‐ignition (LOI) results from the surface sediments of the study lakes in the Wapusk National Park region. Table S4. List of the diatoms taxa identified in surficial sediments of the study lakes in the Wapusk National Park region. [file ECE3-6-4526-s001.pdf]

Supporting information

Table S1. Summary of the statistical tests performed to assess if water chemistry variables differed among lakes of the three ecozones of the Wapusk National Park region.

Variable	Test	F or K statistic	**p-value**	Post-hoc test	Pairwise comparisons	**p-value**
NO_3	Kruskal-Wallis	4.855	0.0883			
					CF-IPP	0.00714
NH_4	One-way ANOVA	5.732	0.00799	Tukey HSD	CF-BSF	0.221
					IPP-BSF	0.698
					CF-IPP	0.902
log TKN	One-way ANOVA	4.308	0.0230	Tukey HSD	CF-BSF	0.0186
					IPP-BSF	0.0604
TP	One-way ANOVA	0.784	0.784			
DOC	Kruskal-Wallis	3.129	0.209			
					CF-IPP	0.000175
DIC	One-way ANOVA	15.080	< 0.0001	Tukey HSD	CF-BSF	0.000863
					IPP-BSF	0.916
					CF-IPP	< 0.0001
pH	Kruskal-Wallis	22.733	< 0.0001	Nemenyi	CF-BSF	0.0190
					IPP-BSF	0.710
					CF-IPP	0.000117
Alk	One-way ANOVA	16.330	< 0.0001	Tukey HSD	CF-BSF	0.000473
					IPP-BSF	0.872
					CF-IPP	< 0.0001
log F	One-way ANOVA	31.590	< 0.0001	Tukey HSD	CF-BSF	< 0.0001
					IPP-BSF	0.0201
					CF-IPP	0.000120
Na	Kruskal-Wallis	22.917	< 0.0001	Nemenyi	CF-BSF	0.00112
					IPP-BSF	0.948
					CF-IPP	0.00296
log Mg	One-way ANOVA	8.097	0.00161	Tukey HSD	CF-BSF	0.0297
					IPP-BSF	0.990
					CF-IPP	< 0.0001
log Cl	One-way ANOVA	25.240	< 0.0001	Tukey HSD	CF-BSF	< 0.0001
					IPP-BSF	0.810
					CF-IPP	0.000157
log K	One-way ANOVA	15.110	< 0.0001	Tukey HSD	CF-BSF	0.000968
					IPP-BSF	0.942
					CF-IPP	< 0.0001
Ca	One-way ANOVA	16.860	< 0.0001	Tukey HSD	CF-BSF	0.000847
					IPP-BSF	0.995
					CF-IPP	0.00355
log SO_4	One-way ANOVA	8.243	0.00146	Tukey HSD	CF-BSF	0.0191
					IPP-BSF	0.996

CF, Coastal Fen; IPP, Interior Peat Plateau; BSF, Boreal Spruce Forest; NO_3, nitrate; NH_4, ammonium; TKN, total kjeldahl nitrogen; TP, total phosphorus; DOC, dissolved organic carbon; DIC, dissolved inorganic carbon; Alk, alkalinity; F, fluorine; Na, sodium; Mg, magnesium; Cl, chlorine; K, potassium; Ca, calcium; SO_4, sulfate.

Table S1 (concluded).

Variable	Test	F or K statistic	**p-value**	Post-hoc test	Pairwise comparisons	**p-value**
log SiO_2	One-way ANOVA	10.320	0.000413	Tukey HSD	CF-IPP	0.000315
					CF-BSF	0.116
					IPP-BSF	0.386
log Cond	One-way ANOVA	17.550	< 0.0001	Tukey HSD	CF-IPP	< 0.0001
					CF-BSF	0.000674
					IPP-BSF	0.995
% OM	Kruskal-Wallis	4.003	0.135			
log % $CaCO_3$	One-way ANOVA	3.626	0.0374	Tukey HSD	CF-IPP	0.115
					CF-BSF	0.0806
					IPP-BSF	0.886
S	Kuskal-Wallis	9.800	0.00745	Nemenyi	CF-IPP	0.00990
					CF-BSF	0.139
					IPP-BSF	0.935
H'	One-way ANOVA	2.918	0.0695			

CF, Coastal Fen; IPP, Interior Peat Plateau; BSF, Boreal Spruce Forest; SiO_2, silica; Cond, conductivity; % OM, organic matter percentage; % $CaCO_3$, calcium carbonate percentage; S, taxonomic richness; H', Shannon diversity index.

Table S2. Values of the environmental variables obtained for the study lakes in the Wapusk National Park region. Values for the water chemistry variables are expressed as July averages for years 2010, 2011 and 2012.

Lakes	Location	Dist (km)	Area (x 10^3 m^2)	NO_3 (µg/L)	NH_4 (ug/L)	TKN (µg/L)	TP (µg/L)	DOC (mg/L)	DIC (mg/L)
CF									
WAP 01	58.39369° N 93.38201° W	21.1	2584.28	14.67	228.00	1444.40	35.00	7.63	17.47
WAP 02	58.38516° N 93.34488° W	18.6	43.14	13.33	190.33	2213.73	41.33	17.60	30.90
WAP 03	58.34298° N 93.27074° W	15.6	≤ 0.70	6.00	173.00	2294.00	38.00	33.00	8.40
WAP 04	58.34131° N 93.26801° W	15.6	≤ 0.70	12.33	138.33	2664.40	49.33	26.00	20.00
WAP 05	58.34223° N 93.26452° W	15.4	≤ 0.70	28.00	204.67	1520.73	27.00	18.77	13.53
WAP 06	58.35090° N 93.23186° W	13.3	48.38	12.33	166.67	2414.73	36.33	21.00	31.37
WAP 07	58.42721° N 93.17816° W	7.8	25.84	12.33	125.67	1258.73	23.67	12.43	27.13
WAP 08	58.40602° N 93.26443° W	13.3	158.24	25.67	293.67	1545.07	47.67	11.70	19.63
WAP 09	58.41423° N 93.30735° W	15.2	123.13	15.33	176.67	1546.40	16.33	14.70	23.43
WAP 10	58.42525° N 93.26773° W	12.7	≤ 0.70	15.00	169.00	2330.00	29.00	25.40	24.00
WAP 11	58.42532° N 93.26598° W	12.5	≤ 0.70	8.00	110.00	1511.00	27.00	20.10	18.70
WAP 12	58.42558° N 93.26891° W	12.7	≤ 0.70	18.50	240.00	3051.50	37.50	37.95	19.20
WAP 13	58.66047° N 93.19432° W	4.6	1336.25	18.00	278.33	634.73	17.67	7.37	14.93
WAP 14	58.62084° N 93.17439° W	3.7	22.10	11.6	191.00	971.60	19.00	10.57	30.33
WAP 15	58.62001° N 93.17101° W	3.6	93.72	13.00	193.00	866.07	26.67	9.07	27.27
WAP 16	58.54066° N 93.16073° W	3.6	47.15	25.00	140.00	783.73	47.33	7.60	19.63
WAP 17	58.56114° N 93.16701° W	3.9	21.41	56.33	147.33	707.73	35.00	9.02	29.60
WAP 18	58.62165° N 93.31750° W	11.9	26.75	68.67	122.33	1101.73	52.67	13.70	26.63
WAP 19	58.70696° N 93.29921° W	8.1	31.84	15.00	159.00	944.73	53.00	11.63	20.63
WAP 20	58.66995° N 93.44365° W	9.7	23.06	12.67	175.00	1700.07	30.33	19.47	23.23
WAP 21	58.66515° N 93.44088° W	10.2	≤ 0.70	14.33	185.67	1931.73	47.00	23.30	36.20
Minimum*	-	3.6	21.41	11.60	122.33	634.73	16.33	7.37	13.53
Maximum*	-	21.1	2584.28	68.67	293.67	2664.40	53.00	26.00	36.20
Median*	-	10.2	45.15	14.67	176.67	1444.40	35.00	12.43	23.43
IPP									
WAP 22	57.96256° N 94.07730° W	75.4	392.61	12.67	202.33	1172.07	55.67	20.87	2.23
WAP 32	57.99007° N 93.45931° W	40.1	≤ 0.70	72.67	243.67	3064.73	62.67	46.00	1.03
WAP 33	58.05161° N 93.53294° W	42.3	12.61	25.00	94.67	983.73	19.00	14.67	6.93
WAP 34	58.04637° N 93.65920° W	49.4	≤ 0.70	7.67	107.00	1575.40	47.00	29.03	0.87
WAP 35	58.04489° N 93.65900° W	49.4	≤ 0.70	9.00	104.33	1190.73	28.00	26.97	0.80
WAP 36	58.04601° N 93.65985° W	49.4	165.52	12.67	136.00	1314.07	27.33	22.50	13.83
WAP 37	58.07802° N 93.66095° W	47.8	1366.13	14.67	74.00	1225.73	29.34	14.70	27.53
WAP 38	58.11877° N 93.55353° W	43.0	126.02	10.33	87.00	1291.10	6.51	16.73	24.90
WAP 39	58.21463° N 93.70755° W	44.7	7613.82	10.33	55.33	986.73	36.33	9.97	14.47
WAP 40	58.36498° N 93.77685° W	42.8	773.66	10.33	58.33	528.07	14.34	7.47	11.40
Minimum	-	40.1	≤ 0.70	7.67	55.33	528.07	6.51	7.47	0.80
Maximum	-	75.4	7613.82	72.67	243.67	3064.73	62.67	46.00	27.53
Median	-	46.3	145.77	11.50	99.50	1208.23	28.67	18.80	9.17
BSF**									
WAP 23	57.83547° N 94.18272° W	84.9	1087.51	200.33	108.00	982.73	48.00	10.90	13.17
WAP 24	57.73882° N 94.00513° W	77.5	98.20	156.67	169.67	669.40	33.00	15.13	3.67
WAP 25	57.70476° N 94.04647° W	80.7	2686.42	21.33	131.33	639.73	37.00	12.57	10.30
WAP 26	57.69803° N 94.11492° W	84.9	177.37	12.67	175.00	617.07	30.33	16.00	6.13
WAP 27	57.61421° N 93.96950° W	79.1	1196.03	12.00	111.00	710.07	39.33	10.80	10.63
Minimum	-	77.5	98.20	12.00	108.00	617.07	30.33	10.80	3.67
Maximum	-	84.9	2686.42	200.33	175.00	982.73	48.00	16.00	13.17
Median	-	80.7	1087.51	21.33	131.33	669.40	37.00	12.57	10.30

* WAP 03, WAP 10, WAP 11, and WAP 12 have been excluded from the calculations because of potential bias due to mid-summer desiccation events during years 2010 and 2012.

** WAP 28 was excluded of the table because of logistical constraints that prevented annual sampling.

CF, Coastal Fen; IPP, Interior Peat Plateau; BSF, Boreal Spruce Forest; Dist, distance from the coast; NO_3, nitrate; NH_4, ammonium; TKN, total kjeldahl nitrogen; TP, total phosphorus; DOC, dissolved organic carbon; DIC, dissolved inorganic carbon.

Table S2 (concluded).

Lakes	pH	Alk (mg/L)	Ca (mg/L)	Na (mg/L)	Cl (mg/L)	Mg (mg/L)	K (mg/L)	F (mg/L)	SO_4 (mg/L)	SiO_2 (mg/L)	Cond (µs/cm)
CF											
WAP 01	8.62	89.72	21.97	10.32	17.06	5.18	0.85	0.07	2.51	1.16	237.00
WAP 02	8.18	172.66	42.41	28.69	65.49	10.56	1.66	0.11	4.03	1.93	421.67
WAP 03	8.75	50.40	20.21	6.34	11.46	3.58	0.01	0.04	2.17	2.56	112.00
WAP 04	8.78	115.30	29.43	9.61	18.01	5.94	0.24	0.11	2.50	2.98	271.00
WAP 05	8.87	79.53	21.63	6.20	10.42	3.67	0.58	0.10	0.66	1.48	198.67
WAP 06	8.35	176.50	47.48	49.38	110.95	9.43	1.74	0.15	4.39	1.55	675.67
WAP 07	8.28	144.48	39.68	80.40	151.18	16.88	2.91	0.14	9.84	0.74	541.67
WAP 08	8.37	102.72	29.32	7.70	18.67	5.01	0.56	0.09	0.96	0.43	296.33
WAP 09	8.44	130.71	29.61	8.88	15.00	5.70	0.99	0.11	2.15	0.87	342.67
WAP 10	8.60	125.82	47.29	12.91	34.69	7.97	0.86	0.11	3.05	5.84	133.00
WAP 11	8.80	98.60	39.26	11.76	33.73	6.09	1.00	0.07	3.07	2.62	222.00
WAP 12	8.63	137.50	51.28	18.09	52.08	8.84	0.90	0.09	3.49	8.77	401.00
WAP 13	8.56	73.64	20.63	7.36	11.99	4.03	0.78	0.09	0.87	0.75	230.67
WAP 14	8.45	164.10	36.23	21.71	34.65	8.70	1.69	0.26	5.36	2.69	489.33
WAP 15	8.32	144.85	34.17	25.08	42.44	9.11	1.69	0.22	3.11	2.03	418.33
WAP 16	8.70	102.98	25.27	27.29	44.46	7.63	1.95	0.23	11.60	1.40	451.33
WAP 17	8.24	171.70	36.72	32.05	46.15	11.32	2.64	0.33	17.12	5.12	617.67
WAP 18	8.42	150.46	30.91	15.33	23.54	10.06	1.35	0.13	5.95	0.81	401.33
WAP 19	8.57	111.62	23.92	23.13	39.19	10.51	1.56	0.16	3.56	1.42	361.33
WAP 20	8.68	138.66	29.33	142.12	247.60	24.74	4.76	0.13	13.83	3.48	1106.67
WAP 21	8.35	201.91	55.69	85.24	458.92	46.34	8.06	0.21	58.42	0.97	1999.00
Minimum*	8.18	73.64	20.63	6.20	10.42	3.67	0.24	0.07	0.66	0.43	198.67
Maximum*	8.87	201.91	55.69	142.12	458.92	46.34	8.06	0.33	58.42	5.12	1999.00
Median*	8.44	138.66	29.61	23.13	39.19	9.11	1.66	0.13	4.03	1.42	418.33
IPP											
WAP 22	7.60	1.43	3.39	1.90	2.95	1.23	0.49	0.06	0.12	0.75	37.33
WAP 32	5.87	0.00	3.59	5.86	10.64	1.77	0.15	0.07	0.20	0.08	78.00
WAP 33	7.08	29.66	12.37	3.54	5.86	2.76	0.34	0.03	0.61	1.04	112.33
WAP 34	5.79	0.00	1.39	2.36	3.06	1.24	0.27	0.04	0.24	0.10	46.33
WAP 35	5.46	0.00	1.12	2.04	3.46	0.86	0.12	0.08	0.36	0.08	34.00
WAP 36	7.53	73.31	15.08	3.39	5.19	8.59	0.53	0.07	1.47	1.81	198.00
WAP 37	7.24	149.72	31.28	7.27	9.51	9.25	0.86	0.06	4.00	0.84	351.00
WAP 38	8.07	134.17	30.67	4.93	8.86	7.41	0.66	0.04	4.88	0.48	327.67
WAP 39	8.27	67.92	15.81	2.18	4.00	5.83	0.44	0.08	1.95	0.18	167.67
WAP 40	8.13	56.36	14.97	1.78	2.29	5.18	0.41	0.02	0.74	0.14	113.33
Minimum	5.46	0.00	1.12	1.78	2.29	0.86	0.12	0.02	0.12	0.08	34.00
Maximum	8.27	149.72	31.28	7.27	10.64	9.25	0.86	0.08	4.88	1.81	351.00
Median	7.39	43.01	13.67	2.88	4.60	3.97	0.43	0.06	0.68	0.33	112.83
BSF**											
WAP 23	8.07	72.10	16.30	3.10	3.4	5.04	0.36	0.04	2.85	0.20	184.00
WAP 24	7.82	7.41	5.56	0.83	1.2	1.64	0.16	0.01	0.07	0.63	43.00
WAP 25	8.14	55.55	15.75	3.22	6.8	4.61	0.35	0.03	2.05	0.45	115.67
WAP 26	7.59	20.35	9.79	2.49	5.7	2.99	0.36	0.03	0.39	1.12	97.00
WAP 27	8.21	42.39	15.04	3.07	4.0	3.89	0.46	0.04	1.01	1.10	141.33
Minimum	7.59	7.41	5.56	0.83	1.20	1.64	0.16	0.01	0.07	0.20	43.00
Maximum	8.21	72.10	16.30	3.22	6.80	5.04	0.46	0.04	2.85	1.12	184.00
Median	8.07	42.39	15.04	3.07	4.00	3.89	0.36	0.03	1.01	0.63	115.67

* WAP 03, WAP 10, WAP 11, and WAP 12 have been excluded from the calculations because potential bias due to mid-summer desiccation events during years 2010 and 2012.

** WAP 28 was excluded of the table because of logistical constraints that prevented annual sampling.

CF, Coastal Fen; IPP, Interior Peat Plateau; BSF, Boreal Spruce Forest; Alk, alkalinity; Ca, calcium; Na, sodium; Cl, chlorine; Mg, magnesium; K, potassium; F, fluorine; SO_4, sulfate; SiO_2, silica; Cond, conductivity.

Table S3. Loss-on-ignition (LOI) results from the surface sediments of the study lakes in the Wapusk National Park region.

Sediment samples	% OM	% $CaCO_3$
CF		
WAP 01	3.38	17.97
WAP 02	76.19	6.24
WAP 03	87.38	1.43
WAP 04	86.81	12.14
WAP 05	89.67	3.03
WAP 06	77.36	15.77
WAP 07	82.48	5.55
WAP 08	75.58	7.93
WAP 09	84.30	2.64
WAP 10	90.24	3.08
WAP 11	87.46	3.61
WAP 12	88.12	3.78
WAP 13	2.45	45.17
WAP 14	12.27	32.91
WAP 15	46.83	44.10
WAP 16	2.48	52.16
WAP 17	10.37	71.50
WAP 18	61.14	9.86
WAP 19	38.42	48.61
WAP 20	40.71	20.96
WAP 21	23.99	30.86
Minimum	2.45	1.43
Maximum	90.24	71.50
Median	75.58	12.14
IPP		
WAP 22	93.36	3.23
WAP 32	93.13	3.12
WAP 33	89.83	4.82
WAP 34	92.51	3.04
WAP 35	91.57	0.91
WAP 36	81.67	4.88
WAP 37	57.59	11.21
WAP 38	65.81	12.85
WAP 39	2.04	28.84
WAP 40	86.07	5.14
Minimum	2.04	0.91
Maximum	93.36	28.84
Median	87.95	4.85
BSF		
WAP 23	73.19	5.65
WAP 24	92.28	3.51
WAP 25	36.73	8.79
WAP 26	88.86	3.80
WAP 27	1.82	1.91
WAP 28	90.91	3.33
Minimum	1.82	1.91
Maximum	92.28	8.79
Median	81.03	3.66

CF, Coastal Fen; IPP, Interior Peat Plateau; BSF, Boreal Spruce, Forest; % OM, organic matter percentage; % $CaCO_3$, calcium carbonate percentage.

Table S4. List of the diatoms taxa identified in surficial sediments of the study lakes in the Wapusk National Park region. The basionym or a synonym is sometimes provided to reduce taxonomic confusion. The number of occurrences corresponds to the number of lakes where the taxon was observed.

#	Name	Basionym or synonym	Number of occurences	Maximum relative abundance (%)
1	***Achnanthidium minutissimum*** (Kützing) Czarneck 1994 sensu lato	***Achnanthes minutissima*** Kützing 1833	29	26.68
2	***Achnanthidium*** sp. 1		3	0.20
3	***Adlafia bryophila*** (Petersen) Moser and Lange-Bertalot in Moser et al. 1998	***Navicula bryophila*** Petersen 1928	12	1.65
4	***Amphipleura kriegeriana*** (Krasske) Hustedt 1954		7	0.79
5	***Amphora copulata*** (Kützing) Schoeman and Archibald 1986		9	1.85
6	***Amphora dusenii*** Brun 1901		2	0.46
7	***Amphora inariensis*** Krammer 1980		2	0.38
8	***Amphora pediculus*** (Kützing) Grunow 1880		4	3.93
9	***Amphora* sp. (cf. *A. edlundii*** Levkov 2009**)**		1	0.18
10	***Aneumastus tusculus*** (Ehrenberg) Mann and Stickle in Round et al. 1990	***Navicula tuscula*** Ehrenberg 1841	14	2.31
11	***Asterionella formosa*** Hassall 1850		2	1.99
12	***Aulacoseira ambigua*** (Grunow) Simonsen 1979		2	1.96
13	***Aulacoseira distans*** (Ehrenberg) Simonsen 1979		1	0.33
14	***Brachysira microcephala*** (Grunow) Compère 1986		15	4.37
15	***Brachysira styriaca*** (Grunow) Ross in Hartley 1986	***Anomoeoneis styriaca*** (Grunow) Hustedt 1930	4	1.75
16	***Brachysira zellensis*** (Grunow) Round and Mann 1981	***Anomoeoneis zellensis*** Patrick and Reimer 1966	3	1.97
17	***Caloneis bacillum*** (Grunow) Cleve 1894		3	0.39
18	***Caloneis fasciata*** Lagerstedt Cleve 1894		2	0.40
19	***Caloneis fusus*** Hamilton and Antoniades in Antoniades et al. 2009		10	1.18
20	***Caloneis silicula*** (Ehrenberg) Cleve 1894		3	0.93
21	***Caloneis tenuis*** (Gregory) Krammer in Krammer and Lange-Bertalot 1985		4	0.79
22	***Caloneis* sp. (cf. *C. silicula*** (Ehrenberg) Cleve 1894**)**		3	0.92
23	***Cavinula cocconeiformis*** (Gregory ex Greville) Mann and Stickle in Round et al. 1990	***Navicula cocconeiformis*** Gregory ex Greville 1856	1	0.23
24	***Cavinula pseudoscutiformis*** (Hustedt) Mann and Stickle in Round et al. 1990	***Navicula pseudoscutiformis*** Hustedt in Pascher 1930	2	0.33
25	***Chamaepinnularia* sp. 1**		2	1.58
26	***Chamaepinnularia* sp. 2**		1	0.39
27	***Cocconeis placentula*** Ehrenberg 1838 sensu lato		6	26.13
28	***Craticula cuspidata*** (Kützing) Mann 1990		5	0.40
29	***Cyclotella* sp. (cf. *C. atomus*** Hustedt 1937**)**		2	0.39
30	***Cymbella botellus*** (Lagerstedt) Schmidt 1896 in Schmidt 1874-1959		2	0.78
31	***Cymbella cleve-eulerae*** Krammer 2002		4	0.40
32	***Cymbella designata*** Krammer 1985		8	0.99
33	***Cymbella proxima*** Reimer in Patrick and Reimer 1975		1	0.39
34	***Cymbella* sp. (cf. *C. hustedtii*** Krasske 1923**)**		4	0.99
35	***Cymbella* sp. 1**		1	0.20
36	***Cymbopleura amphicephala*** (Naegeli) Krammer 2003	***Cymbella amphicephala*** Naegeli in Kützing 1849	7	1.18
37	***Cymbopleura angustata*** (Smith) Krammer 2003	***Cymbella angustata*** (Smith) Cleve 1894	15	1.76
38	***Cymbopleura elliptica*** Krammer 2003		1	0.39
39	***Cymbopleura hybrida*** (Grunow) Krammer 2003	***Cymbella hybrida*** Grunow in Cleve and Möller 1878	5	1.48
40	***Cymbopleura incerta*** (Grunow) Krammer 2003	***Cymbella incerta*** (Grunow) Cleve 1894	5	0.79

Table S4 (continued).

#	Name	Basionym or synonym	Number of occurences	Maximum relative abundance (%)
41	***Cymbopleura incertiformis* var. *laterostrata*** Krammer 2003		12	1.39
42	***Cymbopleura oblongata*** Krammer 2003		11	1.57
43	***Cymbopleura stauroneiformis*** Krammer 2003	***Cymbella stauroneiformis*** Lagerstedt 1873	3	0.77
44	***Cymbopleura subaequalis*** (Grunow) Krammer 2003	***Cymbella subaequalis*** Grunow in Van Heurck 1880	1	0.37
45	***Cymbopleura* sp. 1**		1	0.39
46	***Denticula tenuis*** Kützing 1844		3	1.19
47	***Diatoma tenuis*** Agardh 1812		23	4.94
48	***Diploneis interrupta*** (Kützing) Cleve 1894		1	0.20
49	***Diploneis marginestriata*** Hustedt 1922		5	1.19
50	***Diploneis parma*** Cleve 1891		1	0.18
51	***Discostella stelligera*** (Cleve and Grunow) Houk and Klee 2004	***Cyclotella stelligera*** Cleve and Grunow 1882	6	1.39
52	***Encyonema fogedii*** Krammer 1997		8	0.55
53	***Encyonema latens*** (Krasske) Mann in Round et al. 1990	***Cymbella latens*** Krasske 1937	1	0.20
54	***Encyonema minutum*** (Hilse) Mann in Round et al. 1990	***Cymbella minuta*** Hilse ex Rabenhorst 1862	1	3.97
55	***Encyonema neogracile*** Krammer 1997		1	2.65
56	***Encyonema norvegicum*** (Grunow) Mayer 1947	***Cymbella norvegica*** Grunow in Schmidt et al. 1875	6	0.79
57	***Encyonema reichardtii*** (Krammer) Mann 1990		3	0.65
58	***Encyonema silesiacum*** (Bleisch) Mann in Round et al. 1990	***Cymbella silesiaca*** Bleisch in Rabenhorst 1864	8	0.93
59	***Encyonema ventricosum*** (Agardh) Grunow 1875	***Cymbella ventricosa*** (Agardh) Agardh 1830	14	1.19
60	***Encyonema* sp. (cf. *E. elginense*** (Krammer) Mann in Round et al. 1990**)**		4	0.79
61	***Encyonema* sp. (cf. *E. fogedii*** Krammer 1997**)**		3	0.79
62	***Encyonema* sp. 1**		1	0.39
63	***Encyonopsis cesatii*** (Rabenhorst) Krammer 1997	***Cymbella cesatii*** Rabenhorst 1881	16	2.16
64	***Encyonopsis descriptiformis*** Bahls 2013		8	0.98
65	***Encyonopsis descripta*** (Hustedt) Krammer 1997	***Cymbella descripta*** (Hustedt) Krammer and Lange-Bertalot 1985	14	2.14
66	***Encyonopsis hustedtii*** Bahls 2013		7	0.79
67	***Encyonopsis subminuta*** Krammer and Reichardt 1997		3	4.37
68	***Encyonopsis* sp. (cf. *Chamaepinnularia soehrensis*** (Krasske) Lange-Bertalot and Krammer in Lange-Bertalot and Metzeltin 1996**)**		6	0.79
69	***Encyonopsis* sp. (cf. *E. falaisensis*** (Grunow) Krammer 1997**)**		1	0.39
70	***Encyonopsis* sp. (cf. *E. hustedtii*** Bahls 2013)		11	1.37
71	***Encyonopsis* sp. (cf. *E. naviculacea*** (Grunow) Krammer 1997/***E. grunowii*** Krammer 1997**)**		4	0.46
72	***Encyonopsis* sp. 1**		1	0.19
73	***Encyonopsis* sp. 2**		2	0.37
74	***Encyonopsis* sp. 3**		11	1.95
75	***Encyonopsis* sp. 4**		2	0.40
76	***Eolimna minima*** (Grunow) Lange-Bertalot and Schiller in Schiller and Lange-bertalot 1997	***Navicula minima*** Grunow in van Heurck 1880	4	12.10
77	***Epithemia adnata*** (Kützing) Brébisson 1838		1	0.23
78	***Epithemia sorex*** Kützing 1844		5	0.98
79	***Epithemia* sp. (cf. *E. adnata*** (Kützing) Brébisson 1838**)**		1	0.20
80	***Epithemia* sp. (cf. *E. argus* var. *alpestris*** (Smith) Grunow 1862**)**		2	0.39
81	***Eucocconeis alpestris*** (Brun) Lange-Bertalot in Lange-Bertalot and Genkal 1999	***Achnanthes flexella* var. *alpestris*** Brun 1880	10	2.84
82	***Eucocconeis flexella*** (Kützing) Meister 1912	***Achnanthes flexella*** (Kützing) Brun 1880	14	1.14
83	***Eucocconeis laevis*** (Østrup) Lange-Bertalot in Lange-Bertalot and Genkal 1999	***Achnanthes laevis*** Østrup 1910	10	1.11
84	***Eunotia arcubus*** Nörpel and Lange-Bertalot in Lange-Bertalot 1993		1	0.38

Table S4 (continued).

#	Name	Basionym or synonym	Number of occurences	Maximum relative abundance (%)
85	***Eunotia arculus*** (Grunow) Lange-Bertalot and Nörpel 1993		1	0.33
86	***Eunotia bilunaris*** (Ehrenberg) Mills 1934 sensu lato		5	4.76
87	***Eunotia fennica*** (Hustedt) Lange-Bertalot 2004		5	9.29
88	***Eunotia incisa*** Smith ex Gregory 1854		3	0.98
89	***Eunotia mucophila*** (Lange-Bertalot, Nörpel, and Alles) Lange-Bertalot in Metzeltin, Lange-Bertalot, and Garcia-Rodriguez 2005	***Eunotia bilunaris* var. *mucophila*** Lange-Bertalot, Nörpel, and Alles in Alles et al. 1991	5	36.17
90	***Eunotia neocompacta* var. *vixcompacta*** Lange-Bertalot in Lange-Bertalot, Bąk, and Witkowski 2011	Both varieties combined and described as ***Eunotia nymanniana*** Grunow in many works (e.g., Krammer and Lange-Bertalot 1991a)	4	27.56
91	***Eunotia neocompacta* var. *neocompacta*** Mayama in Mayama and Kawashima 1998		2	1.19
92	***Eunotia paludosa*** Grunow 1862		3	1.18
93	***Eunotia praerupta*** Ehrenberg 1843 sensu lato		8	3.22
94	***Eunotia rhomboidea*** Hustedt 1950		1	0.20
95	***Eunotia tenella*** (Grunow) Hustedt in Schmidt et al. 1913		2	0.79
96	***Eunotia* sp. (cf. *E. subarcuatoides*** Alles, Nörpel, and Lange-Bertalot 1991**)**		2	1.66
97	***Eunotia* sp. 1**		1	0.37
98	***Eunotia* sp. 2**		2	0.40
99	***Eunotia* sp. 3**		3	0.39
100	***Eunotia* sp. 4**		1	0.20
101	***Eunotia* sp. 5**		1	0.33
102	***Fallacia subhamulata*** (Grunow) Mann in Round et al. 1990	***Navicula subhamulata*** Grunow in Van Heurck 1880	1	0.38
103	***Fragilaria capucina*** Desmazières 1925		27	16.57
104	***Fragilaria capucina* var. *rumpens*** (Kützing) Lange-Bertalot 1991		3	9.27
105	***Fragilaria nanana*** Lange-Bertalot 1991		6	1.29
106	***Fragilaria perminuta*** (Grunow) Lange-Bertalot 2004		7	1.78
107	***Fragilaria tenera*** (Smith) Lange-Bertalot 1980		18	3.75
108	***Fragilaria* sp. (cf. *F. perminuta*** (Grunow) Lange-Bertalot 2004**)**		*11*	*5.74*
109	***Fragilaria* sp. 1**		3	1.78
110	***Fragilaria* sp. 2**		3	2.38
111	***Fragilaria* sp. 3**		10	0.79
112	***Fragilaria* sp. 4**		1	0.20
113	***Fragilariforma constricta*** (Ehrenberg) Williams and Round 1988	***Fragilaria constricta*** Ehrenberg 1843	1	0.40
114	***Fragilariforma constricta* F. *stricta*** (Cleve) Hartley et al. 1996	***Fragilaria constricta* F. *stricta*** Cleve 1895	1	0.79
115	***Fragilariforma exigua*** (Grunow) Kelly	***Fragilaria exigua*** Grunow in Cleve and Möller 1878	1	0.79
116	***Fragilariforma neoproducta*** (Lange-Bertalot) Williams and Round	***Fragilaria neoproducta*** Lange-Bertalot 1991	1	0.33
117	***Fragilariforma* sp. (cf. *F. exigua*** (Grunow) Kelly**)**		1	0.19
118	***Frustulia crassinervia*** (Brébisson) Lange-Bertalot and Krammer in Lange-Bertalot and Metzeltin 1996	***Frustulia rhomboides* var. *crassinervia*** (Brébisson) Ross 1947	2	3.75
119	***Geissleria acceptata*** (Hustedt) Lange-Bertalot and Metzeltin 1996	***Navicula acceptata*** Hustedt 1950	1	0.98
120	***Gomphonema* sp. (cf. *G. gracile*** Ehrenberg 1838**)**		1	0.66
121	***Gomphonema* sp. (cf. *G. minusculum*** Krasske**)**		1	0.40
122	***Gomphonema* sp. (cf. *G. minutum*** (Agardh) Agardh 1831**)**		4	1.15
123	***Gomphonema* sp. 1**		1	0.38
124	***Gomphonema* sp. 2**		1	0.23
125	***Gomphonema* sp. 3**		1	0.39
126	***Gomphonema* sp. 4**		5	0.76

Table S4 (continued).

#	Name	Basionym or synonym	Number of occurences	Maximum relative abundance (%)
127	***Halamphora oligotraphenta*** (Lange-Bertalot) Levkov 2009	***Amphora oligotraphenta*** Lange-Bertalot in Lange-Bertalot and Metzeltin 1996	10	1.76
128	***Hantzschia amphioxys*** (Ehrenberg) Grunow in Cleve & Grunow 1880		1	0.38
129	***Hippodonta hungarica*** (Grunow) Lange-Bertalot, Metzeltin, and Witkowski 1996	***Navicula hungarica*** Grunow 1860	2	1.11
130	***Humidophila* sp. (cf. *H. brekkaensis*** (Petersen) Lowe, Kociolek, Johansen, Van de Vijver, Lange-Bertalot, and Kopalová 2014**)**	***Navicula brekkaensis*** Petersen 1928	5	0.79
131	***Karayevia clevei*** (Grunow) Bukhtiyarova 1999	***Achnanthes clevei*** Grunow in Cleve et Grunow 1880	2	0.65
132	***Karayevia laterostrata*** (Hustedt) Bukhtiyarova 1999	***Achnanthes laterostrata*** Hustedt 1933	1	0.98
133	***Kobayasiella jaagii*** (Meister) Lange-Bertalot 1999	***Navicula jaagii*** Meister 1934	4	0.39
134	***Kobayasiella parasubtilissima*** (Kobayasi & Nagumo) Lange-Bertalot 1999	***Navicula parasubstilissima*** Kobaysi et Nagumo 1988; ***Navicula subtilissima*** as described in Krammer and Lange-Bertalot 1986	4	18.93
135	***Lindavia antiqua*** (Smith) Nakov et al. 2015	***Cyclotella antiqua*** Smith 1853	1	0.20
136	***Lindavia michiganiana*** (Skvortzow) T.Nakov et al. 2015	***Cyclotella michiganiana*** Skvortzov 1937	6	0.78
137	***Mastogloia grevillei*** Smith 1856		3	0.79
138	***Mastogloia lacustris*** (Grunow) Van Heurck 1880	***Mastogloia smithii* var. *lacustris*** Grunow 1878	4	1.97
139	***Navicula aurora*** Sovereign 1958		1	0.20
140	***Navicula caroliniae*** Bahls 2012		2	0.40
141	***Navicula cryptocephala*** Kützing 1844		25	6.28
142	***Navicula digitoconvergens*** Lange-Bertalot in Lange-Bertalot and Genkal 1999		2	0.20
143	***Navicula gregaria*** Donkin 1861		1	0.39
144	***Navicula hintzii*** Lange-Bertalot 1993		1	0.57
145	***Navicula pseudolanceolata*** Lange-Bertalot 1980		2	0.20
146	***Navicula radiosa*** Kützing 1844		12	1.19
147	***Navicula tridentula*** Krasske 1923		3	0.38
148	***Navicula trivialis*** Lange-Bertalot 1980		9	1.71
149	***Navicula vaneei*** Lange-Bertalot in Witkowski, Lange-Bertalot and Stachura 1998		1	0.40
150	***Navicula vulpina*** Kützing 1844		23	5.50
151	***Navicula* sp. (cf. *N. angusta*** Grunow 1860**)**		2	0.39
152	***Navicula* sp. (cf. *N. cryptotenella*** Lange-Bertalot in Krammer and Lange-Bertalot 1985**)**		23	4.03
153	***Navicula* sp. (cf. *N. heimansioides*** Lange-Bertalot 1993**)**		1	0.39
154	***Navicula* sp. (cf. *N. libonensis*** Schoemann 1970**)**		7	1.17
155	***Navicula* sp. (cf. *N. wygaschii*** Lange-Bertalot 2001**)**		15	1.65
156	***Navicula* sp. 1**		1	0.19
157	***Navicula* sp. 2**		1	0.33
158	***Navicula* sp. 3**		3	0.20
159	***Navicula* sp. 4**		1	0.39
160	***Navicula* sp. 5**		3	0.57
161	***Navicula* sp. 6**		2	0.39
162	***Neidium bisulcatum*** (Lagerstedt) Cleve 1894		1	0.38
163	***Neidium dubium*** (Ehrenberg) Cleve 1894		1	0.37
164	***Neidium temperei*** Reimer 1959		1	0.46
165	***Neidium* sp. (cf. *N. ampliatum*** (Ehrenberg) Krammer 1985**)**		1	0.79
166	***Neidium* sp. (cf. *N. bisulcatum*** (Lagerstedt) Cleve 1894**)**		5	0.59
167	***Nitzschia alpina*** Hustedt 1943 emend. Lange-Bertalot 1980		16	3.52

Table S4 (continued).

#	Name	Basionym or synonym	Number of occurences	Maximum relative abundance (%)
168	***Nitzschia angustata*** (Smith) Grunow in Cleve and Grunow 1880		5	0.79
169	***Nitzschia bryophila*** (Hustedt) Hustedt 1943		4	1.19
170	***Nitzschia denticula*** Grunow in Cleve and Grunow 1880	***Denticula kuetzingii*** Grunow 1862	23	53.17
171	***Nitzschia dissipata* var. *media*** (Hantzsch) Grunow in van Heurck 1881		2	0.40
172	***Nitzschia fonticola*** Grunow in Cleve and Möller 1879		4	6.76
173	***Nitzschia gracilis*** Hantzsch 1860		5	7.54
174	***Nitzschia inconspicua*** Grunow 1862		3	0.39
175	***Nitzschia linearis*** Smith 1853		4	0.23
176	***Nitzschia palea*** (Kützing) Smith 1856		23	4.56
177	***Nitzschia perminuta*** (Grunow) Peragallo 1903		28	7.59
178	***Nitzschia recta*** Hantzsch ex Rabenhorst 1862		2	0.38
179	***Nitzschia valdestriata*** Aleem and Hustedt 1951		3	3.36
180	***Nitzschia* sp. (cf. *N. pumila*** Hustedt 1954**)**		1	0.37
181	***Nitzschia* sp. 1**		3	0.38
182	***Nitzschia* sp. 2**		1	2.19
183	***Nitzschia* sp. 3**		1	0.79
184	***Pinnularia biceps*** Gregory 1856		1	0.20
185	***Pinnularia borealis* var. *sublinearis*** Krammer 2000		1	0.33
186	***Pinnularia divergens*** Smith 1853		3	0.38
187	***Pinnularia grunowii*** Krammer 2000		1	0.69
188	***Pinnularia microstauron* var. *nonfasciata*** Krammer 2000		1	0.66
189	***Pinnularia perinterrupta*** Krammer 2000		2	0.96
190	***Pinnularia* sp. (cf. *P. genkalii*** Krammer & Lange-Bertalot in K. Kramer 2000**)**		2	0.20
191	***Pinnularia* sp. 1**		1	0.33
192	***Pinnularia* sp. 2**		6	0.66
193	***Placoneis clementioides*** (Hustedt) Cox 1988	***Navicula clementioides*** Hustedt 1944	1	0.37
194	***Placoneis explanata*** (Hustedt) Lange-Bertalot in Rumrich et al. 2000	***Navicula explanata*** Hustedt 1948	6	1.39
195	***Planothidium frequentissimum*** (Lange-Bertalot) Lange-Bertalot 1999	***Achnanthes lanceolata* subsp. *frequentissima* L**ange-Bertalot 1991	2	4.52
196	***Planothidium oestrupii*** (Cleve) Round and Bukhtiyarova 1996	***Achnanthes oestrupii*** (Cleve-Euler) Hustedt 1930	1	1.77
197	***Planothidium peragalloi*** (Brun and Héribaud) Round and Bukhtiyarova 1996	***Achnanthes peragalli*** Brun and Héribaud 1893	1	0.39
198	***Platessa* sp. (cf. *P. conspicuum*** (Mayer) Aboal in Aboal et al. 2003**)**		1	1.96
199	***Psammothidium helveticum*** (Hustedt) Bukhtiyarova and Round 2007		5	0.92
200	***Psammothidium ventrale*** (Krasske) Bukhtiyarova and Round 1996	Achnanthes ventralis (Krasske) Lange-Bertalot in Krammer and Lange-Bertalot 1991	4	1.19
201	***Psammothidium* sp. 1**		1	0.40
202	***Psammothidium* sp. 2**		2	0.99
203	***Psammothidium* sp. 3**		3	0.38
204	***Pseudostaurosira brevistriata*** (Grunow) Williams and Round 1987	***Fragilaria brevistriata*** Grunow in Van Heurck 1885	15	9.68
205	***Pseudostaurosira brevistriata* var. *papillosa*** (Cleve-Euler) Zimmermann, Poulin, and Pienitz 2010	***Fragilaria brevistriata* var. *papillosa*** Cleve-Euler 1953	1	0.20
206	***Pseudostaurosira* sp. (cf. *P. polonica*** (Witak and Lange-Bertalot) Morales and Edlund in Morales and Edlund 2003**)**		1	0.20
207	***Pseudostaurosira* sp. 1**		6	37.23
208	***Pseudostaurosira* sp. 2**		1	0.78

Table S4 (concluded).

#	Name	Basionym or synonym	Number of occurences	Maximum relative abundance (%)
209	***Reimeria sinuata*** (Gregory) Kociolek and Stoermer 1987	***Cymbella sinuata*** Gregory 1856	1	0.20
210	***Rhopalodia gibba*** (Ehrenberg) Müller 1895		3	0.39
211	***Rossithidium petersenii*** (Hustedt) Aboal in Aboal et al. 2003	***Achnanthes petersenii*** Hustedt 1937	15	12.91
212	***Rossithidium pusillum*** (Grunow) Round and Bukhtiyarova 1996	***Achnanthes pusilla*** (Grunow) De Toni 1891	18	36.63
213	***Sellaphora bacillum*** (Ehrenberg) Mann 1989		4	0.37
214	***Sellaphora mutata*** (Krasske) Lange-Bertalot in Lange-Bertalot et al. 1996	***Navicula pupula* var. *mutata*** (Krasske) Hustedt 1930	1	0.40
215	***Sellaphora pupula*** (Kützing) Mereschkovsky 1902	***Navicula pupula*** Kützing 1844	12	3.24
216	***Sellaphora rectangularis*** (Gregory) Lange-Bertalot and Metzeltin 1996	***Navicula pupula* var. *rectangularis*** (Gregory) Cleve and Grunow 1880	10	0.59
217	***Sellaphora pupula* sp. (cf. *S. meridionalis*** Potapova and Ponader 2008**)**		1	0.38
218	***Stauroneis agrestis*** Petersen 1915		1	0.33
219	***Stauroneis gracilis*** Ehrenberg 1843		4	0.38
220	***Stauroneis reichardtii*** Lange-Bertalot, Cavacini, Tagliaventi, and Alfinito 2003		1	0.69
221	***Stauroneis sagitta*** Cleve 1881	***Stauroneis smithii* var. *sagitta*** (Cleve) Hustedt 1959	1	0.78
222	***Stauroneis siberica*** (Grunow) Lange-Bertalot and Krammer in Lange-Bertalot and Metzeltin 1996	***Stauroneis anceps* var. *siberica*** Grunow in Cleve and Grunow 1880	3	0.39
223	***Stauroneis subgracilis*** Lange-Bertalot and Krammer in Lange-Bertalot and Genkal 1999		1	0.40
224	***Stauroneis* sp. (cf. *S. crassula*** Van de Vijver and Lange-Bertalot 2004**)**		1	0.46
225	***Stauroneis* sp. (cf. *S. fluminopsis*** Van de Vijver and Lange-Bertalot 2004**)**		1	0.78
226	***Stauroneis* sp. (cf. *S. gracilior*** Reichardt 1995**)**		1	0.20
227	***Staurosira construens*** Ehrenberg 1843	***Fragilaria construens*** (Ehrenberg) Grunow 1862	1	0.20
228	***Staurosira construens* var. *binodis*** (Ehrenberg) Hamilton 1992	***Fragilaria construens*** var. ***binodis*** (Ehrenberg) Grunow 1862	2	0.20
229	***Staurosira venter*** (Ehrenberg) Kobayasi 2002	***Fragilaria construens* F. *venter*** (Ehrenberg) Hustedt 1957	7	35.48
230	***Staurosirella pinnata*** (Ehrenberg) Williams and Round 1987	***Fragilaria pinnata*** Ehrenberg 1843	21	66.21
231	***Staurosirella pinnata* var. *acuminata*** Mayer 1937	***Fragilaria pinnata* var. *acuminata*** Mayer 1937	1	0.20
232	***Surirella linearis*** Smith 1853		1	0.74
233	***Tabellaria fenestrata*** (Lyngbye) Kützking 1844		1	0.33
234	***Tabellaria flocculosa*** (Roth) Kützing 1844		17	23.61
235	***Tabellaria flocculosa* var. *linearis*** Koppen 1975		16	3.41
